# Successful wound healing after multiple courses of Rheocarna therapy in a dialysis-dependent chronic limb-threatening ischemia patient: Emphasizing long-term feasibility

**DOI:** 10.1016/j.jvscit.2025.101984

**Published:** 2025-09-12

**Authors:** Akinori Satake, Takahiro Tokuda, Tetsuya Amano

**Affiliations:** Department of Cardiology, Narita Memorial Hospital, Toyohashi, Aichi, Japan; Department of Cardiology, Nagoya Heart Center, Nagoya, Aichi, Japan; Department of Cardiology, Aichi Medical University, Nagakute, Aichi, Japan

The primary goal of treating wound-related chronic limb-threatening ischemia (CLTI) is to achieve wound healing and avoid major amputation. For limb salvage in patients with CLTI, revascularization with bypass surgery or endovascular therapy is recommended.^1^ However, especially in dialysis-dependent patients, achieving successful revascularization can be technically challenging and often unsuccessful.^2^ Therefore, adjunctive therapy is essential for patients with difficult revascularization.

Apheresis is an adjunct treatment option. Rheocarna (Kaneka Corporation) is an apheresis device designed to remove low-density lipoprotein and fibrinogen, thereby improving microcirculation.^2^, ^3^, ^4^ Although its short-term use in CLTI has been documented, its long-term feasibility and tolerability across multiple treatment courses remains underexplored.

We report the case of a 67-year-old male dialysis patient who presented with a nonhealing plantar ulcer (Fig, *A*). Angiography revealed posterior tibial artery occlusion (Fig, *B*). We attempted endovascular therapy for the posterior tibial artery; however, it failed to restore adequate blood flow. Subsequently, Rheocarna therapy was initiated on nondialysis days, three times per week. Four courses of Rheocarna therapy were performed at 2-month intervals, with a maximum of 24 sessions per course. The wound completely healed after the four courses (Fig, *C*). After each course, angiography confirmed the improvement in microcirculation. (Fig, *D*-*G*). The patient tolerated all courses well, with no adverse events, suggesting that long-term, repeated Rheocarna therapy is feasible and may provide substantial benefit in dialysis-dependent CLTI patients without revascularization options.

**Fig:**
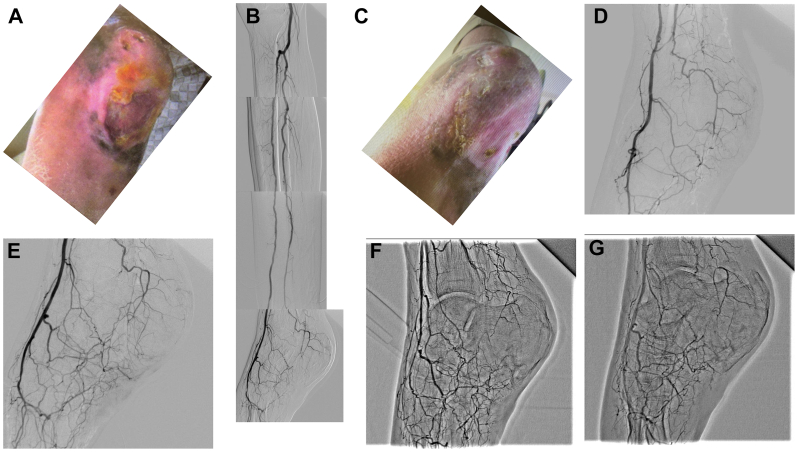
**(A)** Wound status before Rheocarna therapy. **(B)** Angiography images before Rheocarna therapy. **(C)** Wound status after four courses of Rheocarna therapy. **(D)** Angiography images after one course of Rheocarna therapy. **(E)** Angiography images after two courses of Rheocarna therapy. **(F)** Angiography images after three courses of Rheocarna therapy. **(G)** Angiography images after four courses of Rheocarna therapy.

To our knowledge, this clinical report is the first to highlight the successful use of Rheocarna across multiple treatment courses. This case underscores its potential as a safe and sustainable adjunctive therapy in complex CLTI cases, especially in patients with limited revascularization options.

## Funding

None.

## Disclosures

None.
